# Supplementary material for: Practitioners’ views on shared decision-making implementation: A qualitative study
Source: PLoS One. 2021 Nov 11;16(11):e0259844. doi: 10.1371/journal.pone.0259844 (PMC8584754; doi:10.1371/journal.pone.0259844)
Supplement: S1 Appendix — (DOCX) [file pone.0259844.s001.docx]

**Appendix**

**Semi-structured interview guide**

1. Could you tell me a little bit about your background and experiences in the clinic?
2. How would you characterize your experiences with patients under traditional decision-making, i.e. before the Center’s shared decision-making (SDM) initiative?
3. When you encountered the idea of SDM, what was your initial reaction or impression?
4. In your experience, how do patients tend to react when they are invited to take part in SDM consultations?
5. Could you tell me about the Center’s development process for SDM and patient decision aids (PDAs)?
   1. Who were the stakeholders who participated in designing the PDAs and SDM training?
   2. What factors did these stakeholders consider important when designing the PDAs and SDM training?
6. How easy or difficult was it in the beginning to apply your knowledge of SDM from training into practice in the actual consultation? What would you say were the biggest challenges and what factors, if any, did you find helpful?
7. Could you tell me about your experiences with using paper PDAs to conduct SDM consultations?
   1. Besides the clinician, who participates in the SDM consultation with the patient and how are their roles defined? (E.g. nurses)
   2. How are PDAs introduced and used during the SDM consultation?
   3. How are the PDAs kept up-to-date and typically who is responsible for maintaining the PDAs?
   4. How do you explain the various treatment risks and side-effects to the patient?
   5. In your experience, how long does a typical SDM consultation last? Do you feel that your SDM consultations take additional time?
8. What was your experience of the effects of SDM on the consultation process and on outcomes?
9. Do you still experience/observe challenges in carrying out the SDM consultations or using the PDAs? What factors do you think would be helpful to you in reducing these challenges?
