# Supplementary material for: Practitioners’ views on shared decision-making implementation: A qualitative study
Source: PLoS One. 2021 Nov 11;16(11):e0259844. doi: 10.1371/journal.pone.0259844 (PMC8584754; doi:10.1371/journal.pone.0259844)
Supplement: S1 File — (DOCX) [file pone.0259844.s002.docx]

**Consolidated criteria for reporting qualitative studies (COREQ): 32-item checklist**

Developed from:

Tong A, Sainsbury P, Craig J. Consolidated criteria for reporting qualitative research (COREQ): a 32-item checklist for interviews and focus groups. *International Journal for Quality in Health Care*. 2007. Volume 19, Number 6: pp. 349 – 357

**YOU MUST PROVIDE A RESPONSE FOR ALL ITEMS. ENTER N/A IF NOT APPLICABLE**

| **No. Item** | **Guide questions/description** | **Reported on Page #** |
| --- | --- | --- |
| **Domain 1: Research team and reﬂexivity** |  |  |
| *Personal Characteristics* |  |  |
| 1. Interviewer/facilitator | Which author/s conducted the interview or focus group? | Page 7, line 103  AA conducted all the interviews. |
| 2. Credentials | What were the researcher’s credentials? E.g. PhD, MD | Pages 6-7, lines 100-103  This study’s researchers’ credentials are as follows: AA (MSc, PhD student), KDS (MD, PhD), KO (BScN), AD (PhD), LW (PhD), CR (PhD), HH (MSc, PhD student), RF (PhD). |
| 3. Occupation | What was their occupation at the time of the study? | Pages 6-7, lines 100-103  AA and HH were PhD students. KDS was practicing as an oncologist and a researcher. KO, AD, LW, CR, and RF were all researchers. |
| 4. Gender | Was the researcher male or female? | N/A:  Six researchers were female and two were male. These details are not reported because the study context is not gender-specific. |
| 5. Experience and training | What experience or training did the researcher have? | Page 7, line 102  AA, KDS, KO, CR, and RF had prior experience in conducting qualitative research. |
| *Relationship with participants* |  |  |
| 6. Relationship established | Was a relationship established prior to study commencement? | Page 7, line 103-110  Prior to this research, KDS and KO had been practicing at the study setting and were acquainted with some of the study participants. AA conducted the interviews and was not acquainted with the study participants prior to the start of the study, apart from initial acquaintance with KDS from whom she obtained approval to visit the clinic and conduct the study. |
| 7. Participant knowledge of the interviewer | What did the participants know about the researcher? e.g. personal goals, reasons for doing the research | Page 7, line 108-110  One of the participants (KDS) was familiar with the study goals before data collection began in order to grant approval. The remaining participants were provided a brief overview via email (by AA) of the purpose and content of the study, data collection procedure, and confidentiality measures. |
| 8. Interviewer characteristics | What characteristics were reported about the interviewer/facilitator? e.g. Bias, assumptions, reasons and interests in the research topic | Page 7, lines 103-110 and Page 11, lines 178-183  The interviewer (AA) was a visiting researcher throughout the duration of the study. As such, she had little exposure to the clinic context and was able to view its SDM implementation from an external perspective. The purpose was to investigate success factors of SDM implementation by exploring practitioners’ perspectives of the barriers and facilitators. KDS granted permission to conduct the research at the clinic and arranged site meetings with SDM training developers, PDA designers, project managers, and other researchers to familiarize AA with the clinic setting. In addition, AA observed KDS’s patient consultations to gain a better understanding of how SDM and PDAs are applied in practice.  When data collection through semi-structured interviewing began, KDS suggested a number of clinicians from different specializations in order to provide a range of perspectives. Through KDS, AA first made the acquaintance of potential participants in person and then followed up via email with a brief outline of the study purpose, procedure, and information on participation. Interviews were conducted at the participants’ office/clinic, which offered a familiar setting.  While it is not possible to completely eliminate interviewer bias, efforts were made to reduce confirmation bias during analysis by having another researcher (HH) independently perform coding on a selection of interviews. This selection included interviews of clinicians, nurses, and a participant in a leadership position so that a range of perspectives were coded.  The potential for leading questions and wording bias was reduced by adapting an interview guide that had been tested on multiple clinicians in a similar study conducted at MAASTRO Clinic. |
| **Domain 2: study design** |  |  |
| *Theoretical framework* |  |  |
| 9. Methodological orientation and Theory | What methodological orientation was stated to underpin the study? e.g. grounded theory, discourse analysis, ethnography, phenomenology, content analysis | Page 11, lines 167-171  Data was analyzed using the thematic analysis method. Transcripts were read line by line in order to gain familiarity with the data, and codes were assigned to fragments of text for each transcript (open coding). These codes were compared across transcripts to ensure consistency. In the second round, the open codes were grouped in categories according to theme (axial coding). These themes were reviewed and rephrased if necessary to reflect their underlying meaning. |
| *Participant selection* |  |  |
| 10. Sampling | How were participants selected? e.g. purposive, convenience, consecutive, snowball | Page 6, lines 99-101  The participants of this study were selected by means of purposive sampling. |
| 11. Method of approach | How were participants approached? e.g. face-to-face, telephone, mail, email | Page 7, lines 108-110  KDS suggested a number of clinicians from different specializations in order to provide a range of perspectives. Through KDS, AA first made the acquaintance of potential participants in person and then followed up via email with a brief outline of the study purpose, procedure, information on participation, and an invitation to participate. |
| 12. Sample size | How many participants were in the study? | Page 8, line 127-128  In total, 10 participants took part in the study (5 oncologists and 5 healthcare professionals with a nursing background). |
| 13. Non-participation | How many people refused to participate or dropped out? Reasons? | Page 10, lines 151-152  All participants who were approached participated There were no participants who declined to participate or dropped out. |
| *Setting* |  |  |
| 14. Setting of data collection | Where was the data collected? e.g. home, clinic, workplace | Page 10, lines 149-151  All the interviews were conducted in the participants’ respective clinics/offices. |
| 15. Presence of non-participants | Was anyone else present besides the participants and researchers? | Page 10, lines 152-153  Only the researcher (AA) and the participant were present in the interviews. |
| 16. Description of sample | What are the important characteristics of the sample? e.g. demographic data, date | Pages 8-9, lines 136-137  Characteristics of the participant sample are provided in Table 1. |
| *Data collection* |  |  |
| 17. Interview guide | Were questions, prompts, guides provided by the authors? Was it pilot tested? | Page 7, lines 111-117  The interview guide was created based on prior findings in the literature on barriers and facilitators to SDM and PDAs as well as prior research at MAASTRO Clinic that explored this theme.  Was it pilot tested?  Yes, the interview guide was based on previous research conducted at MAASTRO Clinic. In this research, oncologists participated in semi-structured interviews to determine the barriers and facilitators for SDM and a PDA, both of which were yet to be implemented. This interview guide was modified to account for the fact that Vejle Hospital had implemented SDM and PDAs, but the focus on barriers and facilitators remained. In keeping with the semi-structured nature of the data collection, minor modifications to the interview guide were also made following the first 2-3 interviews. This was to allow participants to expand more on aspects of the implementation that they found relevant. |
| 18. Repeat interviews | Were repeat interviews carried out? If yes, how many? | N/A  No repeat interviews were conducted. |
| 19. Audio/visual recording | Did the research use audio or visual recording to collect the data? | Page 10, line 153  All the interviews were audio-recorded. |
| 20. Field notes | Were ﬁeld notes made during and/or after the interview or focus group? | Page 11, lines 162-165  Field notes were made during initial informational meetings with researchers, SDM training developers, PDA developers, and other staff, as well as during the observation of patient consultations. Additional notes were made immediately after each interview and contained the interviewer’s reflections about the interview’s content and specific issues brought up by the participant. |
| 21. Duration | What was the duration of the interviews or focus group? | Page 10, lines 153-155  All the interviews were audio-recorded and lasted on average 49 minutes (range: 30-71 minutes). |
| 22. Data saturation | Was data saturation discussed? | Page 10, lines 160-161  Data collection was stopped when no new themes emerged from the participants’ responses, as per the coding framework that was being constructed in parallel. |
| 23. Transcripts returned | Were transcripts returned to participants for comment and/or correction? | Page 7, lines 156-167  Yes, transcripts were returned to participants for their review and they were given the opportunity to make clarifications or corrections. |
| **Domain 3: analysis and ﬁndings** |  |  |
| *Data analysis* |  |  |
| 24. Number of data coders | How many data coders coded the data? | Page 11, lines 173-177  AA coded all the transcripts. Independently, HH coded three of the transcripts. AA and HH then systematically compared their codes for each text fragment and discussed their interpretations. |
| 25. Description of the coding tree | Did authors provide a description of the coding tree? | Page 13, line 195  Figure 1 presents the themes in tabular form. |
| 26. Derivation of themes | Were themes identiﬁed in advance or derived from the data? | Page 11, lines 171-172  Themes were derived from the data. |
| 27. Software | What software, if applicable, was used to manage the data? | Page 11, lines 175-177  The interviews were transcribed verbatim and saved as Word documents. The coding was performed on these Word documents and then codes were then compiled into an Excel document for further categorization. |
| 28. Participant checking | Did participants provide feedback on the ﬁndings? | As researchers involved in SDM implementation, KDS and KO provided feedback on the analysis. |
| *Reporting* |  |  |
| 29. Quotations presented | Were participant quotations presented to illustrate the themes/ﬁndings? Was each quotation identiﬁed? e.g. participant number | Page 12-20 (Results section)  Relevant quotes have been provided in the Results section. Each quote is labelled with the participant number. |
| 30. Data and ﬁndings consistent | Was there consistency between the data presented and the ﬁndings? | Pages 12-20 (Results section)  Yes, there was consistency between the data presented and the findings. See Results section. |
| 31. Clarity of major themes | Were major themes clearly presented in the ﬁndings? | Page 12, lines 193-194  Yes. The major themes affecting clinician motivation were: mindset, format of the PDA, clinician training in SDM, ownership, and organizational culture and leadership. |
| 32. Clarity of minor themes | Is there a description of diverse cases or discussion of minor themes? | N/A. No minor themes or diverse cases emerged from the data analysis. |

**Once you have completed this checklist, please save a copy and upload it as part of your submission. When requested to do so as part of the upload process, please select the file type: *Checklist*. You will NOT be able to proceed with submission unless the checklist has been uploaded. Please DO NOT** **include this checklist as part of the main manuscript document. It must be uploaded as a separate file.**
